# The Effect of Deep Sedation with High Flow Nasal Oxygen Therapy on the Transcutaneous CO_2_ and Mitochondrial Oxygenation: A Single-Center Observational Study

**DOI:** 10.3390/s25247573

**Published:** 2025-12-13

**Authors:** Annika M. van Smaalen, Calvin J. de Wijs, Sanne E. Hoeks, Egbert G. Mik, Floor A. Harms

**Affiliations:** 1 Laboratory of Experimental Anesthesiology, Department of Anesthesiology, Erasmus MC University Medical Center Rotterdam, 3015 GD Rotterdam, The Netherlands; 2 Department of Anesthesia, Erasmus MC University Medical Center Rotterdam, 3015 GD Rotterdam, The Netherlands

**Keywords:** deep sedation, transcutaneous CO_2_, mitochondrial oxygenation, non-invasive monitoring, wearable sensors

## Abstract

Deep Sedation (DS) allows for shorter recovery times, reduced complication rates and increased cost-effectiveness compared to general anesthesia. In prolonged DS, High Flow Nasal Oxygen Therapy (HFNOT) ensures adequate oxygenation. Concerns remain regarding potential masking of inadequate ventilation and induction of hyperoxia. In this single-center observational study, we continuously monitored tcPCO_2_ and mitoPO_2_ in 30 patients using the SenTec Monitoring System and Cellular Oxygen METabolism (COMET^®^, Photonics Healthcare, Utrecht, The Netherlands) device to observe the effect of prolonged DS with HFNOT on periprocedural ventilation and oxygenation. Measurements were taken at baseline and 30, 60, 90 and 120 min after starting DS. tcPCO_2_ significantly increased after 30 (55.5 (34.5–61.9) mmHg, *p* < 0.001), 60 (54.8 (52.5–62.2) mmHg, *p* < 0.001), 90 (56.5 (53.1–69.3), *p* < 0.001) and 120 (55.8 (50.7–56.6) mmHg, *p* = 0.02) minutes of DS compared to baseline (37.3 (34.5–45.5) mmHg), surpassing the normal range (35–45 mmHg). mitoPO_2_ increased non-significantly from baseline (69.6 (43.9–76.7) mmHg) compared to 30 (80.5 (65.7–98.9) mmHg, *p* = 0.19), 60 (78.6 (70.3–85.8) mmHg, *p* = 0.19), 90 (74.4 (52.7–86.3) mmHg, *p* = 0.38) and 120 (85.6 (82.5–98.0) mmHg, *p* = 0.38) minutes. We observed increased tcPCO_2_ and a non-significant rise in mitoPO_2_ over time, without adverse effects. These findings highlight the potential of continuous sensor-based monitoring to improve real-time detection of ventilation and oxygenation.

## 1. Introduction

Procedural sedation induces a state of reduced consciousness, providing pain control and minimizing recollection [1]. The American Society of Anesthesiologists categorizes sedation into four levels based depth [2]. In moderate sedation, patients respond to verbal or tactile stimuli, whilst in deep sedation, responsiveness is limited to repeated or painful stimuli (Ramsay Score of 5–6). Deep Sedation (DS) involves greater ventilation suppression than moderate sedation [3]. Unlike general anesthesia, DS does not require intubation, with manual techniques like a jaw thrust or Mayo tube usually sufficing [2]. Sedation strategies depend on procedure duration and patient preferences. Dutch guidelines recommend propofol over midazolam for longer procedures requiring moderate to deep sedation [4]. DS offers several advantages over general anesthesia in minimally invasive procedures, including reduced recovery times, improved hemodynamic stability and minimized risk of nasopharyngeal damage [5]. Moreover, DS may have a lower carbon footprint and a reduced climate change score when compared to general anesthesia [6]. In the Netherlands, nurse anesthetists can become sedation practitioners after completing a one-year program, enabling them to independently perform DS under the supervision of an anesthesiologist, thereby improving cost-efficiency [4].

High Flow Nasal Oxygen Therapy (HFNOT) delivers heated and humidified air via a nasal canula at flow up to 70 L/min, with inspiratory oxygen fraction (FiO_2_) up to 1.0, therefore minimizing hypoxic events during DS [7,8]. While it enhances patient comfort and improves alveolar ventilation [9], high FiO_2_ could potentially induce hyperoxic vasoconstriction, reducing microvascular perfusion despite supranormal arterial oxygen levels. Hyperoxia has also been associated with increased oxidative stress and impairment of endothelial function [10,11]. However, its effects at the tissue level remain largely unknown, as current monitoring systems measure oxygenation in different physiological compartments, such as arterial or venous blood, rather than directly at the microcirculatory or cellular level.

A recently developed parameter to assess tissue oxygenation is the mitochondrial oxygen tension (mitoPO_2_), measured non-invasively and in real-time using the Cellular Oxygen METabolism (COMET^®^) device. Mitochondria are the end user of oxygen, and mitoPO_2_ therefore represents the balance between oxygen delivery and consumption at cellular level, offering direct insight into oxygen availability for ATP synthesis [12,13]. Conventional systemic parameters such as SpO_2_ and PaO_2_ remain essential, but they provide limited information about microcirculatory perfusion or cellular oxygen utilization. SpO_2_ rapidly reaches a plateau at high FiO_2_ levels, making it insensitive to further increases or decreases in tissue oxygenation. As high FiO_2_ is administered continuously during DS, evaluating mitochondrial oxygenation could potentially help detect whether hyperoxygenation adversely affects tissue oxygen delivery or microcirculatory dynamics. Moreover, PaO_2_ does not account for local changes in perfusion, metabolic demand, or oxygen extraction, whereas mitoPO_2_ can detect such discrepancies because it reflects cellular oxygenation, and can reveal tissue hypoxia or impaired oxygen utilization even when blood oxygen levels appear normal [14,15]. Increases in mitoPO_2_ can result from increased oxygen delivery (e.g., increased perfusion or partial pressure of arterial oxygen (PaO_2_)) or reduced consumption (e.g., due to low metabolic demand or mitochondrial dysfunction). Because of this dual dependency, mitoPO_2_ interpretation is context-dependent and ideally supported by simultaneous measurements of mitochondrial oxygen consumption (mitoVO_2_) [16].

While HFNOT optimizes oxygenation during DS, high FiO_2_ may mask hypoventilation, increasing the risks of hypercapnia. Capnography has been shown to reduce the incidence of hypoxic events during DS by enabling early detection of hypoventilation [17]. End-tidal CO_2_ (etCO_2_) monitoring, which relies on exhaled air, is less accurate in DS due to dilution by ambient air. Transcutaneous CO_2_ (tcPCO_2_) monitoring via the SenTec Monitoring System overcomes this by measuring CO_2_ directly through the skin and therefore offering potential novel insights into the CO_2_ dynamics during DS. Moreover, this method has been validated as a reliable reflection of PaCO_2_ during DS by several studies [18,19,20,21].

The primary objective of this study was to assess the effect of DS with HFNOT on ventilation and tissue oxygenation, as measured by tcPCO_2_ and mitoPO_2_. The secondary objectives encompassed the effect of time on tcPCO_2_ and mitoPO_2_, length of hospital stay, and adverse events, as well as the effect of DS with HFNOT on the mitoVO_2_. Lastly, this study explores the feasibility and potential clinical value of combining these technologies during prolonged DS.

## 2. Materials and Methods

In this single-center observational trial at the Erasmus Medical Center (Erasmus MC) Rotterdam, The Netherlands, we continuously and intraoperatively monitored tcPCO_2_ and mitoPO_2_ in patients undergoing DS with HFNOT (Fisher & Paykel Healthcare, Auckland, New Zealand). To be eligible for participation, patients had to be at least 18 years of age, scheduled for a procedure of at least 90 min requiring DS with HFNOT and have sufficient proficiency of the Dutch language. The exclusion criteria included a diagnosis of porphyria, known intolerance to components of the ALA-plaster, mitochondrial disease, pregnancy or lactation, skin lesions at the measurement location that could impede measurements and inability to provide informed consent.

A measurement was considered successful if the duration of DS with HFNOT and the monitoring period for tcPCO_2_ and mitoPO_2_ each exceeded 30 min. The stipulated minimum duration of 30 min was chosen to align with the statistical analysis plan, aiming to facilitate a comparison between baseline and those taken 30 min thereafter. Additionally, intraoperative monitoring data had to be available. Unsuccessful measurements were excluded from the analysis.

In December 2022, approval was granted by the Medical Ethics Committee of the Erasmus MC, Rotterdam, The Netherlands (MEC-2022–0421), and the trial was registered in the Clinical Trial Registry (NCT06124027). All participating patients provided written informed consent prior to any research-related activities. Patients in whom measurements were unfeasible due to logistical constraints or who withdrew their informed consent, were subsequently replaced. This manuscript adheres to the STROBE-guidelines [22].

### 2.1. Perioperative Management

Standard monitoring during DS included (SpO_2_), electrocardiogram (ECG), and non-invasive blood pressure (NIBP). Additionally, measurements of tcPCO_2_ and mitoPO_2_ were incorporated for the purpose of this study. Preoperatively, HFNOT was initiated, with flow rates adjusted according to the patient’s needs based on SpO_2_ and clinical manifestations, which were continuously monitored by a sedation practitioner. SpO_2_ levels were maintained above 93%. The FiO_2_ delivered by the HFNOT devices was fixed at 100%, as the devices available did not allow for alteration of the FiO_2_. Sedation induction was performed with propofol (0.4–1.0 mg/kg). Maintenance of anesthesia was achieved via a propofol infusion (4–6 mg/kg/h) and remifentanil infusion (2–4 µg/kg/h), aiming to maintain a Ramsay Score of 5–6. Blood pressure support was provided with noradrenaline as needed.

### 2.2. tcPCO_2_ Measurements

tcPCO_2_ was measured continuously at one-second intervals throughout the procedure using the SenTec Digital Monitoring System (SenTec AG, Therwil, Switzerland). By placing a heated sensor on the forehead, a hyperemic dermis is established resulting in the dilation of local capillaries. This hyperemia-driven vasodilation amplifies arterial blood flow, thereby enhancing the diffusion of CO_2_ through the skin, as measured by the sensor [23]. As was previously described, several studies validated tcPCO_2_ to be a reliable reflection of the PaCO_2_, as opposed to the etCO_2_ [18,19,20,21].

### 2.3. mitoPO_2_ Measurements

mitoPO_2_ was measured at one-minute intervals throughout the procedure using the non-invasive Cellular Oxygen METabolism (COMET^®^, Photonics Healthcare, Utrecht, The Netherlands) device, which utilizes the protoporphyrin IX Triplet State Lifetime technique (PpIX-TSLT). The PpIX-TLST employs oxygen-dependent quenching of PpIX, which acts as a mitochondrial located oxygen-sensitive dye after photo-excitation with a pulse of green light. The delayed fluorescence of protoporphyrin IX is inversely related to the available oxygen concentration in the mitochondria [24]. Detailed descriptions of background principles of the PpIX-TLST can be found elsewhere [12,13,25]. The PpIX-TLST has been validated and calibrated in healthy volunteers [26], and its feasibility has been demonstrated in the operating theatre [27,28], the intensive care unit [29,30] and patients with peripheral arterial disease undergoing endovascular therapy [31].

### 2.4. mitoVO_2_ Measurements

The mitoVO_2_ measurements are based on dynamic mitoPO_2_ (1 Hz) measurements, during which local pressure is applied to the measurement probe for 60 s, inducing reproducible stop-flow conditions in the microcirculation. Halting the oxygen supply while mitochondria continue to consume oxygen, results in a time-dependent reduction in mitoPO_2_. This enables the direct measurement of the oxygen disappearance rate, thereby providing quantifiable mitoVO_2_ measurements. The calculation of mitoVO_2_ is based on an adapted Michaelis-Menten fit procedure, which also accounts for the diffusion of oxygen back into the measurement volume [16]. This analysis results in the calculation of the maximal oxygen consumption (V_max_) in mmHg s^−1^. In order to consistently calculate the mitoVO_2_, the Automated V_max_ Calculation COMET^®^ Application (Erasmus MC, Rotterdam, The Netherlands) was utilized [32]. mitoVO_2_ was measured approximately 5 min before the start and end of DS.

### 2.5. Preparation and After Care

The PpIX-TLST requires an upregulated concentration of PpIX [33]. Therefore, 8 mg of 5-aminolevulinic acid (ALA), a precursor of PpIX, was topically applied as a plaster (Alacare, Photonamic GmbH und Co. KG, Pinneberg, Germany) on both upper arms at least four hours before the measurement. Preparation of the plaster site involved shaving and gentle removal of the top layer of the stratum corneum with a fine abrasive pad. The mitoPO_2_ measurements were performed at the plaster sites. After the measurement, the sites were covered with a light-shielding plaster (Aluderm^®^ plaster, W.Söhngen GmbH, Taunusstein, Germany) to protect against sun exposure for at least 24 h. The day after the procedure, the patient was contacted to inquire whether they had experienced side effects caused by the plasters.

### 2.6. Baseline Characteristics and Intra- and Postoperative Data

Baseline characteristics, medical history, medication use, and intraoperative measurements were extracted from electronic patient records. Furthermore, electronic patient records were reviewed 30 days post-procedure to document the length of hospital stay (mean ± SD) and number of (serious) adverse events. Adverse events encompass any unfavorable occurrences experienced by a participating patient throughout the study, irrespective of the association with the measurements. A serious adverse event is any medical occurrence resulting in death, being life-threatening, requiring hospitalization or prolonging it, causing persistent or significant disability, or any other important medical event based on researcher judgment.

### 2.7. Sample Size

A clinically significant difference between baseline tcPCO_2_ and 30 min after start of DS based on clinical expertise was established at 1.33 kPa (9.975 mmHg). The mean standard deviation, derived from a study examining the superiority of tcPCO_2_ over end-tidal CO_2_ measurements in non-intubated patients [20], was 11.6 mmHg (1.55 kPa). Using G*power [34] and a paired Wilcoxon signed-rank test, an effect size of 0.86 was calculated based on the clinically significant difference and the mean standard deviation. To achieve 95% power at a significance level of 0.01 with an effect size of 0.86, the required sample size was calculated to be 29. To account for potential measurements failures and participant attrition, the sample size was increased by 20%, resulting in a total of 35 patients.

### 2.8. Statistical Analysis

Statistical analyses were performed using R Statistical Software version 4.3.1 (R Foundation for Statistical Computing, Vienna, Austria) [35]. Distributions of standard hemodynamic and respiratory parameters, as well as the incidence of complications, were subjected to descriptive statistical analysis. The normality of continuous variables was assessed with the Shapiro–Wilk test. Normally distributed data were reported as mean ± standard deviation, while non-normally distributed data were reported as median (interquartile range). Categorical variables were summarized as frequencies and percentages. An alpha of 0.05 was considered significant for all analyses.

The paired Wilcoxon-signed rank test was used to compare tcPCO_2_ and mitoPO_2_ measurements at baseline with those 30, 60, 90 and 120 min after starting DS. The time points represent the average of measurements taken over 5 min intervals: 30 min (30–35 min), 60 min (60–65 min), 90 min (90–95 min), and 120 min (120–125 min) after starting DS. To account for multiple testing, the Benjamini–Hochberg correction method was used [36]. Baseline mitoPO_2_ was defined as the mean of the first 30 measurements at a 1 s interval before the interval was set to one measurement per minute. Baseline tcPCO_2_ was defined as the first 30 measurements after sensor calibration.

A linear mixed model with a random effects structure was fitted to investigate the effect of HFNOT with DS on the tcPCO_2_ and mitoPO_2_ over time. Depending on the level of non-linearity of the data, natural splines with three knots were added to improve the model fit. The ANOVA test was used to select the best-fitting model. Assumptions of the linear mixed model were tested, including an examination of the residuals.

## 3. Results

**Figure 1:**
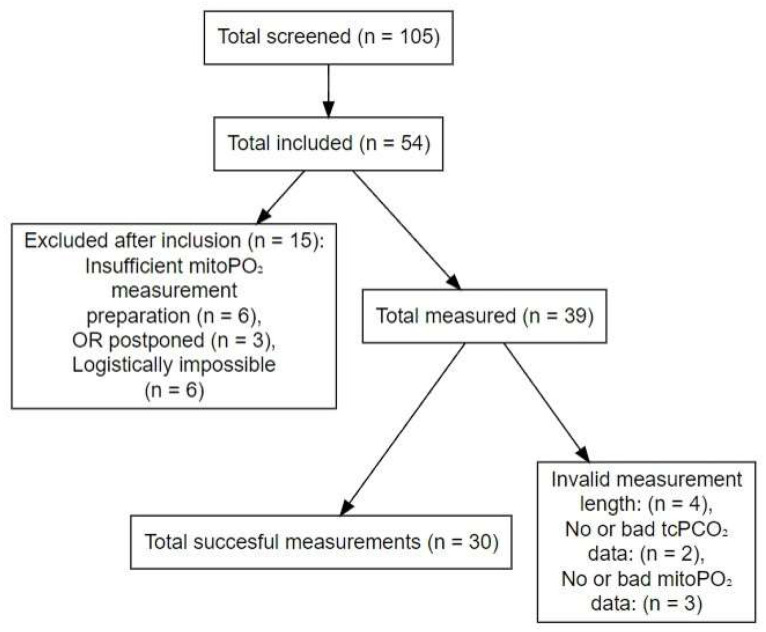
Flowchart of inclusion process. Abbreviations: tcPCO_2_, transcutaneous CO_2_; mitoPO_2_, mitochondrial oxygenation; OR, operation room.

In total, 105 patients were screened for eligibility, and 54 patients were included. Of these, 25 were excluded before the measurements due to withdrawal of informed consent, insufficient preparation for mitoPO_2_ measurements or logistical difficulties (Figure 1).

Between February and November 2023, measurements were conducted in 39 patients, of which 30 were successful. In four patients, measurements were considered unsuccessful according to the aforementioned criteria. Additionally, two patients had no or bad quality tcPCO_2_ data, and three patients had no or bad quality mitoPO_2_ data (Figure 1). All patients completed the follow-up.

### 3.1. Patient Characteristics

Baseline characteristics are shown in Table 1. The study sample included 30 patients, (21 males and 9 females), with a mean age of 60.0 ± 12.0. The median DS time was 89 (61–106) minutes, and the median HFNOT time was 80 (63–111) minutes. The median tcPCO_2_ measurement time was 96 (63–116) minutes, and the median mitoPO_2_ measurement time was 104 (71–116) minutes. The median SpO_2_ was 98% (96–99). All patients underwent interventional radiologic or endoscopic procedures. Oncologic disease was the most prevalent comorbidity (76.7%). The median length of hospital stay was 1 (0–1.8) day. Two minor adverse events occurred: a skin abrasion from the preparation for mitoPO_2_ measurements and a case of desaturation during transport, managed with oxygen therapy. Subsequent follow-up revealed no additional complications associated with these adverse events. No serious adverse events were reported during 30-day follow-up.

### 3.2. Effect of DS with HFNOT on tcPCO_2_

Data was analyzed up to 120 min as only five patients underwent DS for a longer period. The median tcPCO_2_ at 30 min was based on 29 patients due to a calibration error in one case. The sample size reduced over time due to procedure completion: 25 patients at 60 min, 16 at 90 min, and 7 at 120 min.

**Figure 2:**
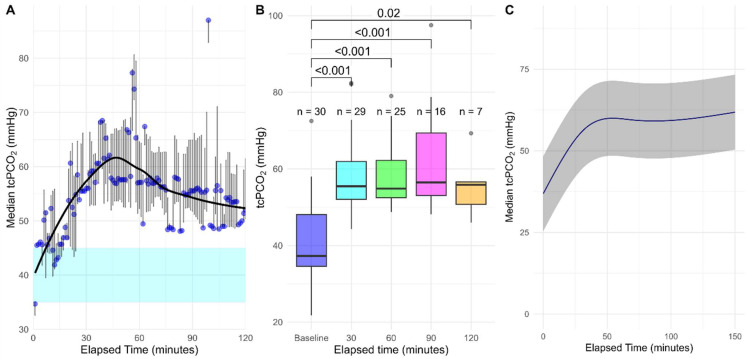
Median tcPCO_2_ in mmHg grouped per minute denoted by the blue dots, with the error bars denoting the interquartile range, the black line denoting the Locally Estimated Scatterplot Smoothing (LOESS) regression line and the blue box denoting the normal range (**A**); baseline tcPCO_2_ in mmHg compared to tcPCO_2_ at 30, 60, 90 and 120 min after start of deep sedation using the paired Wilcoxon signed-rank (**B**); effect plot of univariable linear mixed model of tcPCO_2_ in mmHg over time with the grey ribbon denoting the 95% confidence interval (**C**). Abbreviations: tcPCO_2_, transcutaneous CO_2_.

Baseline tcPCO_2_ was 37.3 (34.5–45.5) mmHg, which significantly increased to 55.5 (34.5–61.9) mmHg after 30 min of DS (*p* < 0.001). This increase remained significant at 60 min (54.8 (52.5–62.2) mmHg, *p* < 0.001), 90 min (56.5 (53.1–69.3) mmHg, *p* < 0.001), and 120 min (55.8 (50.7–56.6) mmHg, *p* = 0.016) (Table 2, Figure 2A,B). The univariable model showed a significant effect of time on tcPCO_2_ (Table 3 and Figure 2C).

### 3.3. Effect of DS with HFNOT on mitoPO_2_

**Figure 3:**
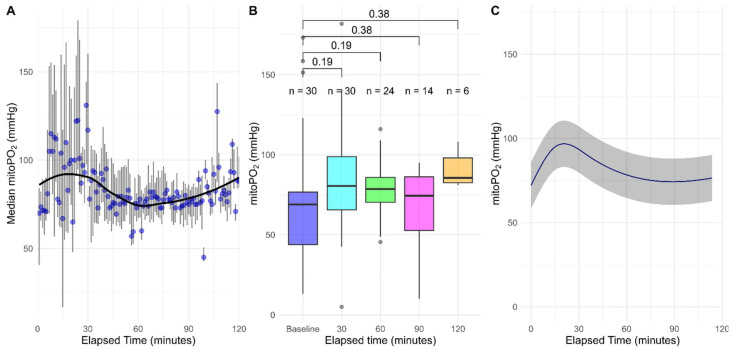
Median mitoPO_2_ in mmHg grouped per minute denoted by the blue dots, with the error bars denoting the interquartile range and the black line denoting the Locally Estimated Scatterplot Smoothing (LOESS) regression line (**A**); baseline mitoPO_2_ in mmHg compared to mitoPO_2_ at 30, 60, 90 and 120 min after start of deep sedation using the paired Wilcoxon signed-rank test (**B**); effect plot of univariable linear mixed model of mitoPO_2_ in mmHg over time with the grey ribbon denoting the 95% confidence interval (**C**). Abbreviations: mitoPO_2_, mitochondrial oxygenation.

Figure 3A shows mitoPO_2_ data up to 120 min. Data from 30 patients was available at baseline and after 30 min of DS, which subsequently decreased over time due to procedure completion: 24 patients after 60 min, 14 after 90 min and 6 after 120 min. Median baseline mitoPO_2_ was 69.6 (43.9–76.7) mmHg, increasing to 80.5 (65.7–98.9) mmHg after 30 min, though not significantly (*p* = 0.19). Similarly, non-significant increases were observed at 60 (78.6 (70.3–85.8) mmHg, *p* = 0.19), 90 (74.42 (52.7–86.3) mmHg, *p* = 0.38), and 120 min (85.6 (82.5–98.0) mmHg, *p* = 0.38) (Table 2, Figure 3B). Lastly, the univariable model showed a significant effect of time on mitoPO_2_ in the first and second spline (Table 3 and Figure 3C).

### 3.4. Effect of DS with HFNOT on mitoVO_2_

The mitoVO_2_ measurement before DS, after DS or both were infeasible in five patients due to logistical restraints. Additionally, in six patients, the analysis software had difficulty processing the data, leading to incomplete measurements. Therefore, the mitoVO_2_ data consists of 19 patients. The median mitoVO_2_ before the start of DS (4.12 (3.47–4.78) mmHg s^−1^) did not significantly increase compared to the mitoVO_2_ five minutes before the end of DS (4.32 (3.40–5.63) mmHg s^−1^, *p* = 0.37), as is presented in Supplemental Table S1 and Supplemental Figure S2.

## 4. Discussion

In this study, we examined the effects of DS with HFNOT on tcPCO_2_ and mitoPO_2_. The analysis revealed a significant increase in the median tcPCO_2_ at all four timepoints compared to baseline, most likely due to decreased respiratory drive after administration of DS induction medication [37,38,39]. This was further reiterated by the mixed model, indicating that tcPCO_2_ increases over time during DS.

In DS, tcPCO_2_ can be used as a viable non-invasive and continuously measured parameter to detect hypercapnia. Aarrestad et al. [18] validated tcPCO_2_ measured with the SenTec Digital Monitoring System as a reliable reflection of PaCO_2_, with no differences exceeding 1 kPa. In another study, prolonged tcPCO_2_ monitoring was also well tolerated, and the correlation between tcPCO_2_ and PaCO_2_ remained highly significant [19]. Similarly, Lermuzeaux et al. [20] found a strong correlation between tcPCO_2_ and PaC O_2_, but a poor correlation between etCO_2_ and PaCO_2_ in non-intubated patients with acute respiratory failure. Lastly, De Oliveira et al. [21] demonstrated that tcPCO_2_ monitoring was more sensitive in detecting hypercapnia than nasal capnography.

Notably, the median tcPCO_2_ exceeded the normal range (35–45 mmHg) at all time points except baseline, indicating prevalent hypercapnia in this cohort. The persistent elevation in tcPCO_2_ may have clinically relevant implications, as hypercapnia can increase intracranial pressure, contribute to respiratory acidosis, and place additional strain on the cardiovascular system [40]. Although hypoxemia was effectively prevented by HFNOT, the high FiO_2_ may mask clinically important hypoventilation [17,41], meaning that hypercapnia could otherwise remain undetected without tcPCO_2_ monitoring. These findings highlight the additional value of tcPCO_2_ as an early-warning parameter during prolonged deep sedation, although further research is required to establish its precise clinical utility. However, no hypercapnia-related adverse events were observed in this cohort, nor was the study designed to detect these events. Future research is required to clarify the exact impact of hypercapnia in DS patients receiving HFNOT.

mitoPO_2_ did not significantly increase from baseline at any time point. HFNOT is expected to increase cellular oxygenation due to the delivery of high concentrations of oxygen; however, this may also trigger hyperoxic vasoconstriction, potentially reducing tissue oxygenation [42,43,44,45]. At the same time, hypercapnia may develop, potentially masked by HFNOT, which could lead to hypercapnic vasodilation, increasing blood flow. This could possibly explain the observed effect, although adequately designed preclinical studies are required to clarify this physiological interaction.

In our study, the median mitoPO_2_ was consistently higher than those reported in other studies using the COMET^®^ device (healthy volunteer studies: 66 ± 16 mmHg [46] and 68 mmHg (61–77) [47]), studies in the operating theatre: 62 ± 23 mmHg [28], 60 ± 19 mmHg [27] and 39 (30 to 50) mmHg [48]). This may be due to the 100% FiO_2_ provided by the HFNOT during DS, which is five times higher than the atmospheric oxygen concentration of 21% to which the healthy volunteers were subjected. Moreover, such high FiO_2_ values are uncommon in the OR as they may increase the risk of long-term mortality [49]. Although mitoPO_2_ values in our study were higher than in previous reports, there are currently no established thresholds for safe or toxic mitoPO_2_ levels. Recent studies in cardiothoracic surgery patients have suggested that mitoPO_2_ values below ~30 mmHg may be associated with an increased risk of postoperative complications, including CSA-AKI and elevated lactate levels [14,28,50]. In our cohort, mitoPO_2_ remained well above this threshold. The absence of adverse events, combined with stable mitoVO_2_, suggests that these supranormal values may not be clinically harmful in this setting. However, this study was not designed to detect these events.

Mitochondrial monitoring may have several potential clinical applications during prolonged DS. Continuous mitoPO_2_ assessment could help detect microcirculatory impairment, hyperoxic vasoconstriction, or declining cellular oxygen availability before alterations appear in systemic parameters. Such early-warning capability may support individualized oxygen titration strategies or inform the need for ventilatory adjustments. Future controlled studies could evaluate whether mitoPO_2_-guided sedation improves outcomes or reduces oxygen-related complications.

The mixed model found a significant initial increase in mitoPO_2_, followed by a decrease and plateau near baseline values. Although pairwise comparisons at individual time points did not reach statistical significance, the spline-based mixed model did reveal a significant effect of time on mitoPO_2_. This may indicate a time-dependent trend in mitochondrial oxygenation during DS with HFNOT, potentially reflecting physiological changes not captured in fixed-time comparisons. It is possible that the high FiO_2_ provided by HFNOT increased mitoPO_2_ initially, but vasoconstriction in response to hyperoxia may have caused the subsequent decline, causing a subsequent decrease in mitoPO_2_. This was also noted in a recent study by Hilderink et al. [45] whom found that hyperoxia resulted in a decrease in mitoPO_2_, likely due to a reduction in microcirculation.

No significant increase in mitoVO_2_ was found, indicating that the oxygen consumption is likely not excessively affected by the high concentrations of oxygen administered. Moreover, this suggests that the observed increase in mitoPO_2_ was more likely attributable to increased oxygen delivery rather than a reduction in mitochondrial oxygen consumption. This aligns with the expected physiological effect of HFNOT delivering high inspired oxygen concentrations.

Both the SenTec Monitoring System and the COMET^®^ device offer the unique advantage of continuous and non-invasive parameter monitoring. Moreover, the SenTec Monitoring system allows for rapid and straightforward application, facilitating the seamless integration of this monitoring modality into perioperative logistics. Besides the plaster preparation, the COMET^®^ Device is convenient in usage and is also easily integrated into perioperative logistics. Plaster preparation is an essential step in the monitoring process and requires extra effort from both the patient being monitored and the assessor. Aside from two minor skin abrasions, no complications regarding the plaster preparation process were reported. Beyond the physiological findings, this study highlights the technological and clinical innovation of integrating the SenTec and COMET^®^ systems into the perioperative workflow. The SenTec Digital Monitoring System enabled continuous, high-resolution tcPCO_2_ monitoring, providing real-time visualization of ventilation dynamics during DS. This underlines its potential as a reliable and non-invasive tool for early identification of hypoventilation during prolonged DS, while seamlessly fitting into existing procedural workflows. The COMET^®^ system represents a novel advancement in clinical monitoring by quantifying mitoPO_2_ in real time. Unlike conventional oxygenation parameters such as SpO_2_ or PaO_2_, which primarily reflect systemic oxygen delivery, mitoPO_2_ provides direct insight into cellular oxygen utilization and microcirculatory dynamics. The ability to measure this parameter non-invasively and continuously introduces a new dimension to perioperative monitoring, bridging the gap between macro- and microcirculatory oxygen assessment.

From an implementation perspective, both systems were easily incorporated into routine sedation workflows with minimal setup time and without interference in procedural logistics. This demonstrates the feasibility of integrating advanced physiological monitoring technologies into daily clinical practice. These modalities have the potential to enhance diagnostic accuracy, optimize oxygen titration, and improve overall patient safety during prolonged DS. Future research should focus on validating these systems in larger, multicenter settings and exploring their potential to guide individualized sedation and oxygenation strategies.

### Strengths & Limitations

This single center observational study allowed for the examination of ventilation and oxygenation during DS procedures with HFNOT in a real-world clinical setting, offering new insights into these parameters and their potential clinical application. A key strength is the continuous, non-invasive monitoring of both tcPCO_2_ and mitoPO_2_. These two parameters are not yet routinely assessed during procedural sedation and provide a more nuanced understanding of ventilation and tissue oxygenation dynamics.

However, this study design does not allow for the investigation of causal relationships because of the observational nature. Moreover, as the study was conducted in a single academic institution where DS is routinely performed using HFNOT with 100% FiO_2_, the generalizability of our findings may be limited. Sedation protocols, including oxygen delivery strategies, can vary substantially between centers. The use of high FiO_2_ during DS is common in many clinical settings to minimize the risk of hypoxemia [51]. Nevertheless, the physiological and clinical effects of lower or titrated FiO_2_ levels during sedation have not been systematically studied. Future multicenter studies are therefore warranted to validate our findings and to explore the impact of different oxygenation strategies on patient outcomes.

Moreover, the observational nature of this study limited our ability to alter standard monitoring protocols during DS. While arterial blood gas (ABG) sampling at multiple intervals could have provided more direct insights into PaCO_2_ and PaO_2_ fluctuations, this approach was deemed too invasive and inconsistent with routine clinical practice. Frequent ABG sampling could also prolong the procedure and cause unnecessary discomfort to patients. Instead, two non-invasive monitoring modalities were added alongside standard monitoring. SpO_2_ levels were maintained above 93% per protocol, ensuring the detection and management of hypoxia or hyperoxia as per standard guidelines.

Sedation practitioners were not blinded to the measured tcPCO_2_ and mitoPO_2_ measurements. To mitigate introduction of behavioral bias, sedation practitioners were advised to discard the observed values. Despite this, unconscious responses may have influenced care, potentially introducing bias.

The median duration of sedation was 89 min, with the longest nearing 4 h. Only a small number of patients underwent sedation for more than 120 min, resulting in limited data for these longer procedures (see Supplemental Figure S1). Consequently, formulating statements regarding extremely prolonged sedation procedures, exceeding 2 h, is challenging due to the infrequency of such events in this study.

The statistical power of this study was determined based on being able to find a significant difference in tcPCO_2_. Consequently, this study may have been underpowered to detect significant differences in mitoPO_2_. A well-powered study is necessary to draw robust conclusions regarding the effect of DS with HFNOT on mitoPO_2_.

No adverse events were observed during the 30-day follow-up. However, the incidence of respiratory complications in DS is generally reported as low [52]. As this study was neither designed nor powered to address safety in terms of adverse event incidence, we cannot adequately assess this based on our data. Although no hyperoxia-related adverse events were observed in this study, the possibility of harm from prolonged exposure to high FiO_2_ cannot be excluded. Therefore, further research is needed to assess the safety of sustained high FiO_2_ levels in relation to hyperoxia and its consequences.

Both the SenTec and COMET^®^ monitoring modalities do have limitations. In this study, tcPCO_2_ measurements failed in two patients. One failure was due to a delayed membrane replacement by the technical service, while in another case, prolonged calibration resulted in insufficient data for analysis. Proper maintenance and user knowledge are crucial for effective use of the SenTec Monitoring System in clinical practice. Similarly, mitoPO_2_ measurements failed in three patients, primarily due to improper adhesive application and malfunctioning of the device due to several instances of COMET^®^ failures related to its sensitive light source. It is noteworthy that the manufacturer acknowledges this limitation and intends to address it in the development of a new version of the COMET^®^ device.

## 5. Conclusions

This study found that tcPCO_2_ levels increased from baseline during prolonged DS with HFNOT. Although mitoPO_2_ showed a tendency to increase over time, this trend was not statistically significant compared to baseline. No significant adverse events attributable to elevated tcPCO_2_ or mitoPO_2_ levels were found. This study also demonstrates the feasibility of continuous, non-invasive monitoring of ventilation and mitochondrial oxygenation using the SenTec and COMET^®^ systems in DS. Both technologies were smoothly integrated into perioperative care, without disrupting workflow.

## Figures and Tables

**Table 1:** Patient characteristics.

	**Overall**		**Overall**
	**(n = 30)**		**(n = 30)**
Male	21 (70.0%)	Diabetes Mellitus	6 (20.0%)
Age (years)	60.0 ± 12.0	Neurodegenerative disease	1 (3.3%)
BMI (kg/m^2^)	27.7 ± 4.8	Cerebrovascular disease	2 (6.7%)
Smoking history	14 (46.7%)	Hypercholesterolemia	1 (3.3%)
Former	12 (40.0%)	Respiratory disease	6 (20.0%)
Current	2 (6.7%)	Cardiovascular disease	15 (50.0%)
Alcohol use	24 (80.0%)	AF	3 (10.0%)
Former	7 (23.3%)	Arrhythmias	2 (6.7%)
Current	16 (53.3%)	DVT	2 (6.7%)
Missing	1 (3.3%)	MI	2 (6.7%)
Respiratory disease	6 (20.0%)	Heart valve disease	1 (3.3%)
OSAS	1 (3.3%)	PVD	1 (3.3%)
COPD	4 (13.3%)	Other	4 (13.3%)
Asthma	1 (3.3%)	Procedure type	
Oncologic disease	23 (76.7%)	Endoscopy	1 (3.3%)
Breast	2 (6.7%)	Colonoscopy	3 (10.0%)
Bladder	1 (3.3%)	Gastro intervention	2 (6.7%)
Colon/rectal	7 (23.3%)	Colon intervention	2 (6.7%)
Renal	4 (13.3%)	RFA kidney	3 (10.0%)
Liver	4 (13.3%)	RFA liver	9 (30.0%)
Lung	1 (3.3%)	TIPS	2 (6.7%)
Prostate	1 (3.3%)	Stenting	4 (13.3%)
Other	3 (10.0%)	Other	4 (13.3%)
Hypertension	16 (53.3%)	High Flow Nasal Oxygen Therapy duration (min)	80 (63–111)
Length of hospital stay (days)	1 (0–1.8)	tcPCO_2_ measurement time (min)	96 (62–116)
Adverse events	2 (6.7%)	mitoPO_2_ measurement time (min)	104 (71–116)
Sedation duration (min)	89 (61–106)		
Oxygen saturation (%)	98 (96–99)		

Data reported as number (proportion), mean ± standard deviation if normally distributed or median (Q1–Q3) if not normally distributed. Abbreviations: Q1: first inter quartile range; Q3, third interquartile range; AF, atrium fibrillation; DVT, deep venous thrombosis; MI, myocardial infarction; PVD, peripheral vascular disease; OSAS, obstructive sleep apnoea syndrome; COPD, chronic obstructive pulmonary disease; RFA, radiofrequency ablation; TIPS, transhepatic intrajugular portosystemic shunt; min, minutes.

**Table 2:** Median tcPCO_2_ and mitoPO_2_ at different timepoints with corresponding inter quartile range and sample size.

Timepoint	Sample Size	Median	Q1–Q3	*p*-Value
tcPCO_2_ (mmHg)				
Baseline	30	37.3	34.5–45.5	
30 min	29	55.5 *	34.5–61.9	<0.001
60 min	25	54.8 *	52.5–62.2	<0.001
90 min	16	56.5 *	53.1–69.3	<0.001
120 min	7	55.8 *	50.7–56.6	0.02
mitoPO_2_ (mmHg)				
Baseline	30	69.6	43.9–76.7	
30 min	30	80.5	65.7–98.9	0.19
60 min	24	78.6	70.3–85.8	0.19
90 min	14	74.4	52.7–86.3	0.38
120 min	6	85.6	82.5–98.0	0.38

* Denotes a significant effect between baseline and the corresponding timepoint. Abbreviations: Q1: first inter quartile range; Q3, third interquartile range; tcPCO_2_, transcutaneous CO_2_; mitoPO_2_, mitochondrial oxygenation; min, minutes; mmHg, millimeters of mercury.

**Table 3:** Univariable mixed model of effect of time on tcPCO_2_ and mitoPO_2_.

	Estimate	Confidence Interval
tcPCO_2_ (mmHg)		
Intercept	36.9	33.5–40.3
Spline (time 1)	11.9 *	11.8–12.1
Spline (time 2)	44.6 *	44.4–44.9
Spline (time 3)	12.3 *	12.1–12.4
mitoPO_2_ (mmHg)		
Intercept	72.1	63.9–80.3
Spline (time 1)	−21.3 *	−25.3–−17.3
Spline (time 2)	30.0 *	25.1–34.9
Spline (time 3)	−2.0	−7.0–2.9

* Denotes a significant effect. Abbreviations: tcPCO_2_, transcutaneous CO_2_; mitoPO_2_, mitochondrial oxygenation; mmHg, millimeters of mercury.

## Data Availability

The raw data supporting the conclusions of this article will be made available on request from the corresponding author as publicly sharing the data was not included in the informed consent form signed by the participants.

## Supplementary Materials

The following supporting information can be downloaded at https://www.mdpi.com/article/10.3390/s25247573/s1, Figure S1: Median tcPCO_2_ over time, grouped per 5 min with the blue box denoting the normal range and the bars denoting the sample size (A), median mitoPO_2_ over time grouped per 5 min and the bars denoting the sample size (B); Figure S2: Mean mitoVO_2_ five minutes before the start and five min before the end of DS compared using the Wilcoxon signed-rank test; Table S1: Median mitoVO_2_ five minutes before the start and five min before the end of DS compared using the Wilcoxon signed-rank test.

## Abbreviations

The following abbreviations are used in this manuscript:

ABG	Arterial blood gas
AF	Atrium fibrillation
ALA	5-aminolevulinic acid
COMET	Cellular Oxygen METabolism
COPD	Chronic obstructive pulmonary disease
DS	Deep Sedation
DVT	Deep venous thrombosis
ECG	Electrocardiogram
etCO_2_	End-tidal CO_2_
FiO_2_	Inspiratory oxygen fraction
HFNOT	High Flow Nasal Oxygen Therapy
IQR	Interquartile range
LOESS	Locally Estimated Scatterplot Smoothing
MI	Myocardial infarction
min	Minutes
mitoPO_2_	Mitochondrial oxygen tension
mitoVO_2_	Mitochondrial oxygen consumption
mmHg	Millimeters of mercury
NIBP	Non-invasive blood pressure
OR	Operation room
OSAS	Obstructive sleep apnea syndrome
PaO_2_	Partial pressure of arterial oxygen
PIF	Patient information
PpIX-TSLT	Protoporphyrin IX Triplet State Lifetime technique
PVD	Peripheral vascular disease
Q_1_	First inter quartile range
Q_3_	Third interquartile range
RFA	Radiofrequency ablation
SD	Standard deviation
SpO_2_	Oxygen saturation
tcPCO_2_	Transcutaneous CO_2_
TIPS	Transhepatic intrajugular portosystemic shunt
V_max_	Maximal oxygen consumption
