# Supplementary material for: The Effect of Deep Sedation with High Flow Nasal Oxygen Therapy on the Transcutaneous CO2 and Mitochondrial Oxygenation: A Single-Center Observational Study
Source: Sensors (Basel). 2025 Dec 13;25(24):7573. doi: 10.3390/s25247573 (PMC12737040; doi:10.3390/s25247573)
Supplement: Supplementary file 1 [file sensors-25-07573-s001.zip › Figure S1.pdf]

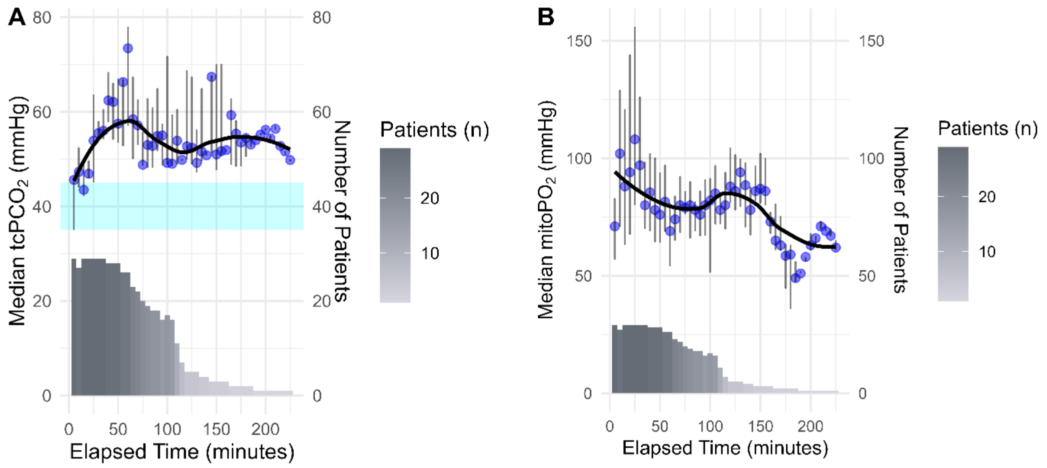

Abbreviations: tcPCO$_2$, transcutaneous CO$_2$; mitoPO$_2$, mitochondrial oxygenation

Supplemental Figure S1. Median tcPCO$_2$ over time, grouped per 5 minutes with the blue box denoting the normal range and the bars denoting the sample size (A), median mitoPO$_2$ over time grouped per 5 minutes and the bars denoting the sample size (B)
