# Supplementary material for: The Effect of Deep Sedation with High Flow Nasal Oxygen Therapy on the Transcutaneous CO2 and Mitochondrial Oxygenation: A Single-Center Observational Study
Source: Sensors (Basel). 2025 Dec 13;25(24):7573. doi: 10.3390/s25247573 (PMC12737040; doi:10.3390/s25247573)
Supplement: Supplementary file 1 [file sensors-25-07573-s001.zip › Figure S2.pdf]

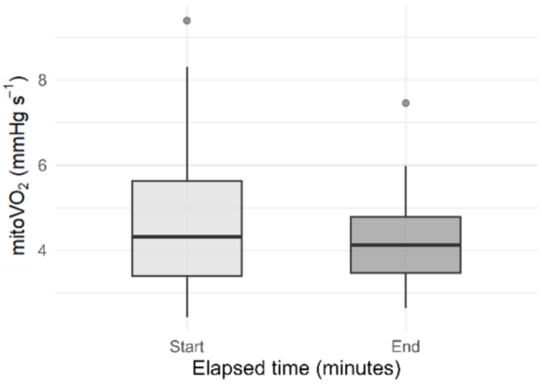

Abbreviations: mitoVO_2, mitochondrial oxygen consumption; mmHg, millimeters of mercury

Supplemental Figure S2. Mean mitoVO_2 five minutes before the start and five minutes before the end of DS compared using the Wilcoxon signed-rank test
